# Is there an optimal age for total knee arthroplasty?: A systematic review

**DOI:** 10.1186/s43019-020-00080-1

**Published:** 2020-11-16

**Authors:** Seung Hoon Lee, Dong Hyun Kim, Yong Seuk Lee

**Affiliations:** 1 Department of Orthopaedic Surgery, Veterans Health Service Medical Center, Seoul, Korea; 2 Department of Orthopaedic Surgery, Seoul National University College of Medicine, Bundang Hospital, Seongnam, Korea

**Keywords:** Total knee arthroplasty, Age, Patient-reported outcome measurement, Revision, Mortality

## Abstract

**Purpose:**

The purpose of this systematic review was to elucidate the optimal age for patients undergoing total knee arthroplasty (TKA), to optimize the balance between the benefits and risks by analyzing patient-reported outcome measurements (PROM), revision rate, and mortality according to age.

**Materials and methods:**

A rigorous and systematic approach was used and each of the selected studies was evaluated for methodological quality. Data were extracted according to the following: study design, patients enrolled, patient age at the time of surgery, follow-up period, PROM, revision rate, and mortality.

**Results:**

Thirty-nine articles were included in the final analysis. The results were inconsistent in the PROM analysis, but there was consensus that PROM were good in patients in their 70s

. In the revision rate analysis, there was consensus that the revision rate tends to increase in TKA in younger patients, but no significant difference was observed in patients > 70 years of age. In the mortality analysis, there was consensus that the mortality was not significantly different in patients < 80 years of age, but tended to increase with age.

**Conclusion:**

This systematic review shows that the PROM were good when TKA was performed in patients between 70 and 80 years of age; the best PROM could be achieved around 70 years of age, and no significant difference in the revision or mortality rates was observed between 70 and 80 years of age; however, mortality tended to increase with age. Therefore, the early 70s could be recommended as an optimal age to undergo TKA.

**Supplementary Information:**

The online version contains supplementary material available at 10.1186/s43019-020-00080-1.

## Introduction

Total knee arthroplasty (TKA) is generally accepted as a cost-effective and successful treatment option for end-stage knee osteoarthritis (OA) [1]. The prevalence of OA is expected to increase in the future and the use of TKA will be expanded along with increased life expectancy, emphasis on quality of life, and implant development. Therefore, there is a possibility that this will result in an increased need for TKA. This also raises the possibility of increased uptake of TKA in younger and older patients (“extreme” age groups) [2, 3]. Therefore, TKA in the extreme age groups could proportionally increase as the volume of TKAs performed increases [4].

TKA can reduce pain and improve patient-reported outcome measures (PROM) and ability to perform activities of daily living. However, TKA can also be accompanied by unexpected complications such as bleeding, acute kidney injury, postoperative delirium, venous thromboembolism, pneumonia, cardiovascular complication, and infection [5–13]. High mortality and morbidity are more frequently observed in older patients [14]. Another important consideration in TKA is the longevity of the implants. Long-term survivorship of a TKA implant up to 20 years after surgery was reported as 97.8% [15]. Considering the average age of the patients, there can be increased need of revision TKA in a younger patient due to aseptic loosening, implant wear, and other reasons related to longevity.

Generally, surgeons are concerned about the outcomes after TKA such as PROM, pain reduction, and patient satisfaction. Moreover, surgeons are also concerned about complications. Risk of revision and mortality are the most important considerations in decision-making when performing TKA. In particular, when performing TKA in the extreme age groups, surgeons are concerned about the risk of revision in younger patients and medical comorbidity and mortality in older patients. In recent studies, it is reported that TKA is a good treatment option for knee OA in the extreme age groups, that is for patients age > 90 years or < 55 years [16–19].

However, the impact of age on patient satisfaction is still debated even though the incidence of TKA uptake among younger (< 55 years) and geriatric (> 80 years) patients is increasing [18, 20]. Some studies have shown good treatment results for TKA even when performed at extreme ages. However, the results of these studies were not analyzed by age [16–18, 21]. Therefore, a better understanding of the effect of age on TKA outcomes, considering the balance between the benefits and risks of TKA, can improve the outcome and facilitate better control of patient expectations [22]. The purpose of this systematic review was to elucidate the optimal age for performing TKA that optimizes the balance between the benefits and risks of TKA, by analyzing PROM results, revision rate, and mortality according to age.

## Materials and methods

### Search strategy

To verify the research question, a rigorous and systematic approach conforming to the preferred reporting items for systematic review and meta-analysis (PRISMA) guidelines was used [23]. In phase 1 of the PRISMA search process, selected databases were searched, including the MEDLINE, EMBASE, and Cochrane database (31 March 2019). This systematic review of the available literature was performed using the keywords: “total knee arthroplasty”, “total knee replacement”, “age factor”, “aged”, “young”, “extreme age”, “old”, “octogenarian”, “nonagenarian”, “treatment outcome”, “revision”, “mortality”, in several combinations. The citations in the included studies were screened, and unpublished articles were also checked with a manual search using Google Scholar. The bibliographies of the relevant articles were subsequently cross-checked for articles not identified in the search. In phase 2, abstracts and titles were screened for relevance. In phase 3, the full text of the selected studies was reviewed according to the inclusion criteria and methodological appropriateness was determined using a predetermined question. In phase 4, the studies were systematically reviewed, if appropriate.

### Eligibility criteria

Studies meeting the following criteria were included: (1) studies on TKA, (2) articles written in English, (3) articles with full text available, (4) human in vivo studies, (5) articles including PROM or revision rate or mortality, and (6) comparative study of results according to age. The exclusion criteria were the following: (1) not related to TKA, 2) no direct comparison according to age category, (3) published before 2000, (4) not a clinical study (review article), (5) TKA not performed for treatment of OA, (6) simultaneous evaluation of TKA and total hip arthroplasty, and (7) not a comparison study by age.

### Data extraction

Each of the selected studies was evaluated for methodological quality by two independent authors. Data were extracted using the following standardized protocol: first author, publication year, publication journal, study type, number of cases, age of the patient at the time of surgery, follow-up period, PROM, revision rate, and mortality, among others. The extracted data were then cross-checked for accuracy, and any disagreements were settled by a third author.

### Quality assessment

The methodological quality of the studies was assessed using the modified Coleman criteria (Additional file 1) [21]. The modified Coleman criteria have a scaled potential score ranging from 0 to 100. Scores of 85–100 are considered excellent, 70–84 good, 55–69 fair, and < 55 poor. The criteria are used to assess the quality of surgical studies.

## Results

### Search

**Fig. 1:**
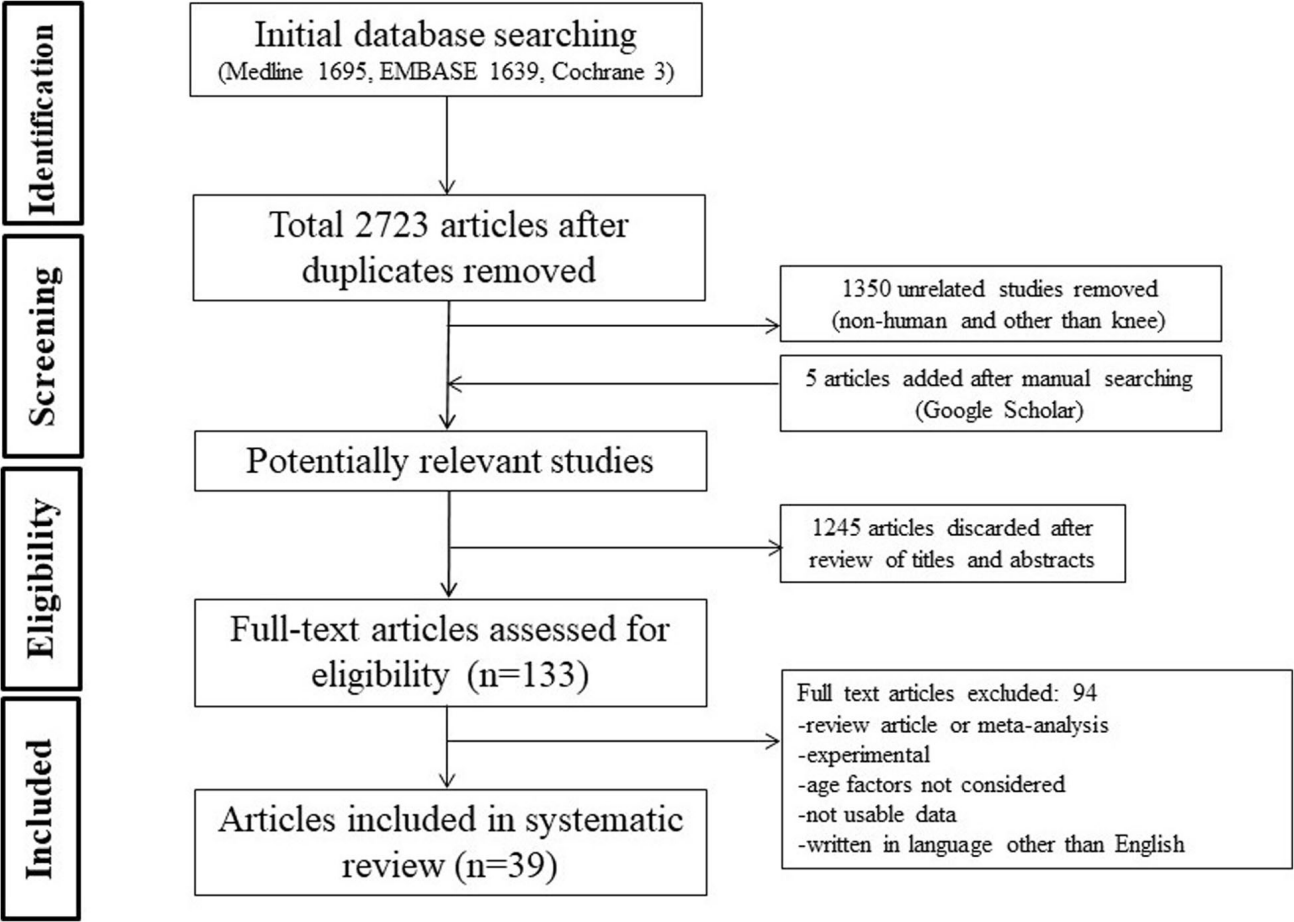
Preferred reporting items for systematic review and meta-analysis (PRISMA) flow chart

The initial electronic search yielded 3337 articles. After removing duplicate studies, and applying the inclusion and exclusion criteria, 39 articles were included in the final analysis. Some articles were studies based on registry data, some involved retrospective cohorts, some enrolled prospective cohorts, and some were case-control studies. The PRISMA flow chart is shown in Fig. 1.

### Quality

The quality of all articles was assessed using the modified Coleman criteria [21]. The studies included and the modified Coleman criteria scores are presented in Table 1. The average modified Coleman criteria score of the studies we analyzed was 56.7, and the scores were good in 3 of the studies, fair in 20 studies, and poor in 16 studies.

**Table 1:** Studies that used the modified Coleman criteria scoring system

Author	Journal	Year	Modified Coleman A score	Modified Coleman B score	Modified Coleman Total Score
Jorgensen [24]	J Bone Joint Surg Am	2019	27	33	60
Pitta [25]	J Arthroplast	2019	20	30	50
Clement [19]	Arch Arthop Trauma Surg	2018	25	30	55
KJ Oh [26]	Aging Clin Exp Res	2018	25	31	56
Lange [27]	J Arthroplast	2018	20	31	51
Murphy [14]	JBJS Rev	2018	17	31	48
Naylor [28]	Arthritis Care Res	2018	27	23	50
Townsend [29]	J Knee Surg	2018	24	28	52
Bayliss [30]	Lancet	2017	29	31	60
Escobar [31]	J Eval Clin Pract	2017	32	28	60
Haynes [32]	Knee	2017	28	27	55
Sveikata [33]	Geriatr Orthop Surg Rehabil	2017	34	26	60
Elmallah [34]	J Knee Surg	2016	32	28	60
Lizaur-Utrilla [35]	Knee Surg Sport Traumatol Arthrosc	2016	31	40	71
Razak [36]	J Bone Joint Surg Am	2016	22	26	48
Shah [20]	J Knee Surg	2016	22	28	50
Skinner [37]	Ann R Coll Surg Eng	2016	22	29	51
Callaghan [38]	Clin Orthop Rel Res	2015	37	33	70
Jauregui [39]	J Arthroplast	2015	21	23	44
Maempel [40]	Acta Orthop	2015	27	33	60
Shin [41]	BMC Musculoskelet Disord	2015	22	28	50
Belmont [42]	J Bone Joint Surg Am	2014	22	30	52
D’Apuzzo [43]	J Arthroplast	2014	22	30	52
Kuo [44]	J Orthop Surg Res	2014	22	33	55
Meehan [45]	J Bone Joint Surg Am	2014	22	31	53
Easterlin [46]	Clin Orthop Rel Res	2013	22	31	53
Hamilton [47]	BMJ Open	2013	29	30	59
Kennedy [48]	Clin Orthop Rel Res	2013	26	33	59
Namba [49]	J Arthroplast	2013	22	38	60
William [50]	Bone Joint J	2013	29	40	69
Jämsen [51]	Acta Orthop	2012	25	32	57
Singh [52]	J Arthroplast	2012	22	28	50
Clement [53]	Bone Joint J	2011	29	38	67
Merle-Vincent [54]	Joint Bone Spine	2011	30	40	70
Wainwright [55]	Bone Joint J	2011	29	35	64
Julin [56]	Acta Orthop	2010	27	33	60
Singh [57]	Osteoarthr Cartil	2010	29	28	57
Robertsson [58]	Bone Joint J	2007	32	29	61
Kreder [59]	J Arthroplast	2005	22	32	54

### PROM

Twenty-two of the studies reviewed provided data based on PROM outcomes. The results were inconsistent and are presented in Table 2. Among the 22 studies, age was not related to PROM in 9 studies but differed according to age in another 9 studies.

**Table 2:** Results for patient-reported outcome measures (PROM)

Author	Journal	Year	Number	Age (years)	Result
***No difference***					
Sveikata [33]	Geriatr Orthop Surg Rehabil	2017	314	< 75, ≥ 75	Post operation 1 year: no difference in pain (*P* = 0.592), stiffness (*P* = 0.729), or function (*P* = 0.082) according to WOMAC and SF-12 physical (*P* = 0.082) and mental (*P* = 0.559) health score
Escobar [31]	J Eval Clin Pract	2017	492		No difference between minimal clinically important difference (MCID)/patient acceptable symptom state (PASS) (*P* = 0.5)
Lizaur-Utrilla [35]	Knee Surg Sport Traumatol Arthrosc	2016	≤ 55: 61 60–70: 61	≤ 55, 60–70	Post operation 5 years, there were no significant differences between groups in KSS knee or function, WOMAC pain or function, or SF-12 physical or mental. However, there were better results in younger patients for KSS function (*P* = 0.018), WOMAC function (*P* = 0.028), SF-12-physical (*P* = 0.001) and SF-12-mental (*P* = 0.035), although clinically relevant
Maempel [40]	Acta Orthop	2015	3144	< 75, 75–80, > 80	Post operation 5 years: all groups showed similar substantial improvements in AKS, which were maintained (all *P* < 0.001)
Kuo [44]	J Orthop Surg Res	2014	1024	< 80, ≥80	Both groups (≥ 80, < 80) had improved in the KSS (≥ 80: 86, < 80: 88), KSFS (≥ 80: 87, < 80: 89), WOMAC (≥ 80: 15.0, < 80: 14.6) scores
Hamilton [47]	BMJ Open	2013	4709		Median age of satisfied group is 70.3 and unsatisfied group is 70.0 (*P* = 0.829)
Kennedy [48]	Clin Orthop Rel Res	2013	≥80: 438 < 80: 2754	< 80, ≥80	There was no difference in pain scores at 3, 5, and 10 years between the ≥ 80 years group and < 80 years group. The KSS was comparable between groups at year 5, but the KSFS was lower in the octogenarians
William [50]	Bone Joint J	2013	2456	< 55 55–64, 65–74, 75–84, ≥ 85	Postoperative scores were comparable across age groups, but a linear trend for greater postoperative improvement in OKS and EQ-5D was seen with decreasing age (*P* < 0.033)
Clement [53]	Bone Joint J	2011	677	< 80, ≥ 80	Post operation 1 year: no significant difference was observed between the groups in the mean improvement in OKS (95% CI − 0.65 to 2.94, *P* = 0.16)
***Difference***					
Pitta [25]	J Arthroplast	2019	3693		For the KOOS pain, KOOS activity, and LEAS outcomes, the divergence point occurred at age 68 years. For the KOOS symptom outcomes, the divergence point occurred at age 70 years.
Elmallah [34]	J Knee Surg	2016	278	< 55, 55–74, > 74	For KSS objective, patiemts 75 years and older had the highest mean score at final follow up (97 points). In KSS function, the < 55-years group had highest scores at 2-year (90 vs. 87 vs. 75 points) and 5-year follow up (96 vs. 88 vs. 72 points). For SF-36 and LEAS, the cohorts 75 years and older had the lowest mean scores at various time points. In the mental component, those < 55 years had the lowest scores postoperatively
***Older better***					
Townsend [29]	J Knee Surg	2018	356	< 50, 50–59, 60–69, 70–79, > 79	Postoperative WOMAC and overall, pain, and function OKS significantly differed among the age groups (*P* < 0.05), with patients younger than 60 years reporting the worst scores in the postoperative time period. Older patients reported better preoperative overall, pain, and function scores and greater post-TKA outcomes than younger patients
Merle-Vincent [54]	Joint Bone Spine	2011	264	≤ 70, > 70,	Age older than 70 years at surgery was associated with a higher satisfaction rate (odds ratio of age ≥ 70 years is 3.9 [1.1–14.3]; *P* = 0.038)
***Younger better***					
Murphy [14]	Bone Joint J	2018	2838	< 80, ≥ 80	SF-12 PCS, coefficient of ≥ 80-group is − 4.46 (− 6.18, − 2.73), *P* < 0.001
KJ Oh [26]	Aging Clin Exp Res	2018	79	65–70, ≥ 80	The octogenarian patient group had significantly inferior outcomes for WOMAC and SF-36 score compared to the sexagenarian patient group (*P* = 0.009 and *P* = 0.022, respectively)
Naylor [28]	Arthritis Care Res	2018	1289		Post operation 3 years: younger age (*P* = 0.0018) was significantly associated with regular physical activity
Razak [36]	J Bone Joint Surg Am	2016	3062		Younger age KSS predicted a good outcome at 5 years (OKS: OR of age is 2.66 (2.61–2.71), SF-36 PCS: OR of age is 2.64 (2.59–2.67))
Singh [57]	Osteoarthr Cartil	2010	7139	61–70, 71–80, > 80	Significantly predictors of overall moderate–severe activity limitation 2-years post-TKA was age 71–80 (OR: 2.1 [1.5, 2.8]) and age > 80 (OR: 4.1 [2.7, 6.1]) vs, age ≤ 60 years, and 5 years post-TKA was age 71–80 (OR: 2.4 [1.7, 3.5]) and age > 80 (OR: 4.7 [2.8, 7.9]) vs. age ≤ 60 years
***Extreme age***					
***Extreme old age***					
Skinner [37]	Ann R Coll Surg Eng	2016	67	70–79, 90–99	For preoperative OKS no significant difference between nonagenarians and control group
***Extreme young age***					
Lange [27]	J Arthroplast	2018	1058	18–55, 65–75	Distribution of satisfaction responses was shifted toward greater satisfaction in older patients (*P* < 0.001). Younger patients reported greater knee-related dysfunction and higher activity levels preoperatively and postoperatively (*P* = 0.0002)
Haynes [32]	Knee	2017	≤ 55: 82 65–75: 85	≤55, 65–75	The younger patient cohort reported substantially lower preoperative clinical outcome scores. WOMAC pain (12.1 points, *P* < 0.01), and WOMAC physical function. (6.9 points, *P* < 0.01) improvement was noted; however, WOMAC pain score remained lower among the younger patient cohort
Clement [19]	Arch Arthop Trauma Surg	2018	2589	< 55, 55 ≤	The younger age group was twice as likely to be dissatisfied with their overall outcome and pain relief, with only 83% and 85% being satisfied compared to 92% and 91% in the older age group, respectively

*KSS* Knee Society Score, *WOMAC* Western Ontario and McMaster Osteoarthritis Index, *KSFS* Knee Society Function Score, *OKS* Oxford Knee Score, OR odds ratio, *SF* Short Form, *PCS* Physical Component Score, *AKS* American Knee Society, *LEAS* Lower Extremity Activity Scale, *ADL* activities of daily living, *TKA* total knee arthroplasty

In studies where no differences in PROM were reported, patients age 75, 80, and 85 years were used as age-related references [33, 35, 40, 44, 48, 50, 53]. PROM were compared in regression analysis in another two studies, and the authors reported no age-related differences [31, 47].

Among the nine studies in which PROM differed according to age, two studies reported that better PROMs were achieved after TKA in older patients [29, 54] and five studies reported that younger patients had better PROM after TKA [26, 28, 36, 57, 60]. However, the baseline age of the patients was 80 years in two of the studies, and in one study the patients’ limitation of activity increased fourfold over the age of 80 years, and it was difficult to compare the differences in PROM in patients age < 80 years. In two studies only, regression analysis showed that physical activity decreased as age increased [28, 36]. Pitta et al. [25] reported that the best PROM was achieved at 68 years of age, and Elmallah et al. [34] reported that the effects of age on Knee Society scores, the Short Form-36 findings, and the lower extremity activity scale were different.

Four studies reported outcomes in the extreme age groups. In the very oldest patients, only one study reported on PROM after TKA: there was no difference between nonagenarians and younger patients in the degree of improvement in PROM [37]. In the very youngest patients, three studies reported PROM after TKA; in all three there was a relatively smaller improvement in clinical outcome in patients < 55 years of age [19, 27, 32].

### Revision rate

Nine studies in this review provided data on revision rates, and the results are presented in Table 3. Eight studies reported that younger patients were more likely to undergo revision until death, and one study reported no difference in revision rates according to age [24, 30, 38, 41, 45, 49, 55, 56].

**Table 3:** Results for revision rate

Author	Journal	Year	Age (years)	Number	Result
***Difference***					
Jorgensen [24]	J Bone Joint Surg Am	2019	< 55, 55–64, 65–74, ≥ 75	478,081	The MAR at 15 years was 3.0% (2.8–3.2%). Age had a significant effect on MAR rates, with cumulative percent revision at 15 years for patients < 55 years old of 7.8% (95% CI, 6.5% to 9.2%) compared with 1.0% for those ≥ 75 years old (95% CI, 0.8% to 1.1%; *P* < 0.001).
Bayliss [30]	Lancet	2017	50–54, 55–59, 60–64, 65–69, 70–74, 75–79, 80–84, ≥85	54,276	For patients aged 70 years at implantation (mean age of implantation) LTRR was between 4·4% and 7·7%. For patients aged between 60 and 70 years, LTRR increased with decreasing age, reaching approximately 15% for both hip and knee replacement at 60 years, with greater risk in male than in female patients. Significant increase in LTRR was seen in younger men, with values 35.0% (30.9–39.1) seen in the youngest patient group (50–54 years)
Shin [41]	BMC Musculoskelet Disord	2015	< 65, 65–74, ≥ 75	260,068	The overall incidence rate of revision TKA was 367.3/100,000 person-years. The incidence in patients 50 years old or younger was extremely high. Incidence rate per 100,000 person-years: < 65 years (447.2), 65–74 years (363.7), ≥ 75 years (270.9)
Callaghan [38]	Clin Orthop Rel Res	2015	< 65, ≥ 65	220	Overall patient survivorship to 20-year follow up was only 26%. Patient survivorship at 20-year follow up was significantly higher in patients < 65 years of age in both cohorts (54% versus 15%, *P* < 0.001 modular tray cohort, and 52% versus 26%, *P* = 0.002 rotating platform cohort).
Meehan [45]	J Bone Joint Surg Am	2014	< 50, 50–64, ≥ 65	120,538	The risk of aseptic mechanical failure was 4.7 times higher (OR = 4.66, 95% CI, 3.77 to 5.76) in patients younger than 50 years of age, 2.1 times higher (OR = 2.09, 90% CI, 1.81–2.41) in patients 50–64 years compared with patients 65 years of age or older
Namba [49]	J Arthroplast	2013	< 65, ≥ 65	64,017	There was a significantly different revision rate (P < 0.001) in the < 65 and ≥ 65 years age groups. For every 10-year increase in age the risk of revision decreases by 38% (95% CI, 33%–43%, *P* < 0.001).
Wainwright [55]	Bone Joint J	2011	< 50, 50–59, 60–75, ≥ 75	1538	Patients younger than 50 years at the time of surgery have a greater chance of requiring revision than of dying, those around 58 years of age have a 50:50 chance of needing revision, and in those older than 62 years the prosthesis will normally outlast the patient.
Julin [56]	Acta Orthop	2010	≤ 55, 56–65, > 65	32,019	The 5-year survival rates were 92% and 95% in patients age ≤ 55 and 56–65 years, compared to 97% in patients who were > 65 years of age (*P* < 0.001) Overall risk of prosthesis failure > 3.7 years follow up: ≤ 55 years (5.0 [3.2–8.0]), 55–65 years (2.0 [1.4–2.9]) vs. > 65 years
***No difference***					
Lizaur-Utrilla [35]	Knee Surg Sport Traumatol Arthrosc	2016	≤ 55: 61 60–70: 61	≤ 55, 60–70	No significant relationship between revision and age younger than 55 and older than 55 years

*CI* confidence interval, *OR* odds ratio, *SD* standard deviation, *MAR* major aseptic revision, *LTRR* lifetime risk of revision

The baseline age of the patients was 65 years in four studies, and high revision rates were reported in the younger group in these studies [38, 41, 45, 49]. Similar results were reported in another study in which the baseline age of the patients was 55 years. Bayliss et al. [30] also reported that the younger age group had higher revision rates, with the lowest implant survival rates seen in patients in their 50s at the time of index surgery and decrease in revision rates seen after 70 years of age. Meehan et al. [45] reported that the revision rate was 4.7 times higher in patients < 50 years of age, and 2.1 times higher in patients 50–64 years of age compared to that noted in patients > 65 years of age. Julin et al. [56] also reported that the revision rate was 5 times higher in patients < 55 years of age and 2 times higher in patients 55–64 years of age compared to that noted in patients > 65 years of age. Wainwright et al. [55] reported that patients < 50 years of age at the time of surgery have a greater chance of requiring revision surgery than of dying, and those around the age of 58 years have a 50:50 chance of requiring revision. In addition to group comparisons, Namda et al. [49] reported a 38% reduction in revision rates with every 10-year increase in age.

Only one study reported no significant difference in revision rates between patients < 55 and ≥ 55 years of age [35]. However, the median follow-up period was 12 years, therefore, the revision rates thereafter could not be confirmed.

### Mortality

Mortality outcomes are presented in Table 4. Fourteen studies in this review provided data on mortality. Among the 14 studies, 12 reported high mortality rates in older patients, whereas 2 studies reported no difference in mortality rates according to age or that younger patients had increased mortality.

**Table 4:** Results for mortality

Author	Journal	Year	Number	Age (years)	Result
***Difference***					
Murphy [14]	Bone Joint J	2018	2838	< 80, ≥ 80	Mortality hazard ratio in ≥ 80-years group is 3.40 (2.54–4.54, *P* < 0.001)
Skinner [37]	Ann R Coll Surg Eng	2016	67	70–79, 90–99	Mortality rates were higher in the nonagenarian group but these were in keeping with the life expectancy projections identified by the Office for National Statistics
Shah [20]	J knee Surg	2016	33,066	< 65, ≥ 65	Young cohort had lower rate of mortality (0.03 vs. 0.18%, *P* < 0.001)
Jauregui [39]	J Arthroplast	2015	35,342	< 90, ≥ 90	Serious postoperative adverse events that were significantly higher in nonagenarians compared to controls included death (0.9% vs. 0.2%; *P* = 0.024)
Maempel [40]	Acta Orthop	2015	3144	< 75, 75–80, > 80	Odds ratios for mortality at 1 year, adjusted for ASA, were 2.2 (1.0–4.5) for age 75–80, and 3.0 (1.3–6.8) for age > 80, relative to age < 75 years
Belmont [42]	J Bone Joint Surg Am	2014	15,321		Patient age (OR = 1.12; 95% CI, 1.06 to 1.17) was independent predictor of mortality
D’Apuzzo [43]	J Arthroplast	2014	5,492,805	< 90, ≥ 90	In-hospital mortality was significantly higher in the older cohort compared to the younger group (2.9% versus 0.2%; *P* < 0.001)
Easterlin [46]	Clin Orthop Rel Res	2013	8950	40–64, 65–69, 70–74, 75–79, 80–84, 85–89	Age was associated with increased risk of mortality starting at age 85 years; mortality in patients 85 years and older was 17 times higher than in those younger than 65 years (OR: 70–74 (1.21), 75–79 (2.85), 80–84 (2.57), 85–89 (17.65)
Kennedy [48]	Clin Orthop Rel Res	2013	≥ 80: 438 < 80: 2754	< 80, ≥ 80	Octogenarians had a higher (*P* < 0.001) mortality rate in Kaplan-Meier survival analysis
Jämsen [51]	Acta Orthop	2012	1998	75–79, 80–84, ≥ 85	Adjusted hazard ratio for age 75–79 years is 1, for 80–84 years it is 1.71 [1.31–2.23], for 85 years or over it is 3.34 (2.39–4.65)
Singh [52]	J Arthroplast	2012	12,484		Older age was associated with higher 90-day all-cause mortality. OR for age (per 5-year increase) is 1.6 (1.3–1.9) in univariate analysis and 1.6 (1.2–1.7) in multivariable-adjusted analysis
Kreder [59]	J Arthroplast	2005	15,029	65–79, > 80	Patients > 80 years of age are 3.4 times more likely to die
***No difference***					
Kuo [44]	J Orthop Surg Res	2014	1024	< 80, ≥ 80	There was no 90-day mortality in either group
Robertsson [58]	Bone Joint J	2007	57,979	< 54, 55–59, 60–64, 65–69, 70–74, 75–79, 80–84, > 85	Patients younger than 55 years had a statistically significant increase in total mortality (standardized mortality ratio: 1.85 [1.53–2.22]) while patients older than 65 years had a statistically significant decrease

*OR* odds ratio

Mortality among patients > 90 years of age was reported in three studies [37, 39, 43]. Two studies reported higher mortality rates in this age group than that in the control group [39, 43], but in another study, mortality rates were higher in the nonagenarian group; however, these were in accordance with life expectancy projections identified by the Office for National Statistics [37].

In four studies, there was an increase in mortality rates with age [42, 52], but there was a sharp rise at around 85 years of age [46, 51]. In three studies, the mortality rate in patients ≥ 80 years of age was higher than that in the control groups [48, 59, 60]. In another study, the mortality rate in patients ≥ 65 years of age was higher than that in patients < 65 years of age [20].

## Discussion

The purpose of this study was to elucidate the optimal age to perform TKA when considering PROM, revision rate, and mortality factors. Based on several studies, the principal findings were as follows: (1) there was no significant difference in the PROM before the age of 80 years, and it is best to perform TKA around 70 years of age; (2) there was no significant difference in the TKA revision rate in patients older than 70 years, but the rate tended to decrease with age; and (3) there was no significant difference in mortality at the age of 80 years, but it tended to increase with age. Therefore, it is considered that TKA should be performed in patients in their early 70s because the PROM would be relatively good, the revision rate would not increase, and the risk of mortality would not be high.

In terms of PROM, the effects of age were inconsistent. Some studies reported that age was not related to PROM, while some reported that older patients have better PROM, and other studies reported the opposite. In addition, some studies reported a relationship between age and PROM, but it was not a linear relationship. However, the age standard was around the 70s in studies that reported that older patients have better PROM and around the 80s in studies that reported that younger patients have better PROM. In a study that found no linear relationship, the best PROM were reported in patients in their 70s. Therefore, there was consensus that PROM were good between 70 and 80 years of age and the best PROM could be achieved in patients in their 70s. Even among the very oldest patients, those > 90 years of age also had good PROM; however, this age range was not considered to be optimal for performing TKA [17, 18]. A previous meta-analysis showed good results even among patients < 55 years of age, but this study did not compare the results according to age.

Most studies were consistent on revision rates, showing mostly that the younger the patient at the time of TKA, the greater is the probability of revision during their lifetime, and most studies compared the revision rate based on 65 or 70 years of age as the reference standard. Therefore, there was consensus that the revision rate tends to increase in younger patients, but there is no significant difference in patients > 70 years of age.

Most studies were consistent on mortality, showing mostly that the older the patient at the time of TKA, the higher is the risk of mortality. Most studies that reported high mortality rates in older patients used the 90s or 80s as the standard age. Only one study compared mortality at the age of 65 years. Some studies did not show an increased mortality rate among these patients in contrast to that in the general population; one study reported that younger patients have higher risk of mortality compared to older patients, but this may be due to selection bias [37, 58]. Therefore, there was consensus that the mortality rate was not significantly different at 80 years of age, but tended to increase with age. In summary, TKA performed between the ages of 70 and 80 years has the best outcome. With respect to mortality, it would be better to perform TKA when the patients are younger. Therefore, the authors of these studies believe that from 70 to 80 years of age is the optimal range for undergoing TKA.

There are many factors that are influenced by age when performing TKA. Older age is the predictive factor for postoperative pneumonia and for postoperative delirium after TKA [8, 61–63]. Some studies report that the risk of infection is high in older patients [45, 64]. The transfusion rate and ICU care are also age-related factors [9, 12, 13, 65]. These complications should also be considered in determining when to perform TKA. In general, however, PROM, revision rate, and mortality are the most common considerations in determining when to perform TKA by considering the patients age. The strength of this study is that the authors only considered patient benefit, revision risk, and mortality when evaluating the outcomes of TKA according to the patient’s age.

This study has several limitations. First, there have been many studies using registry data; however, only some small cohort or comparative study was included. However, the results of these studies were mostly consistent. Second, meta-analysis was not performed due to differences in the age-related criteria used in the studies. Nonetheless, this did not influence the results significantly, because we did not analyze the exact age, but the age-related trends. Third, the possibility of errors due to different follow-up duration and PROM measurement indices in each study cannot be ignored. Fifth, life expectancy differs in each country, and thus, comparison of the results based on specific country may not be possible. Furthermore, we did not take into account the increase in life expectancy, which is another limitation of this study.

## Conclusion

This systematic review shows that the PROM were good when TKA was performed in patients between 70 and 80 years of age; the best PROM could be achieved around 70 years of age, and no significant difference in the revision rate and mortality rate was observed between 70 and 80 years of age; however, mortality after TKA tended to increase with age. Therefore, the early 70s could be recommended as an optimal age to undergo TKA.

## Supplementary Information

**Additional file 1.** Modified Coleman Criteria used of quality assessment of studies.

## Data Availability

All data generated or analyzed during this study are included in this published article.

## Abbreviations

PROM: Patient-reported outcome measures
TKA: Total knee arthroplasty
