# Supplementary material for: Is there an optimal age for total knee arthroplasty?: A systematic review
Source: Knee Surg Relat Res. 2020 Nov 16;32:60. doi: 10.1186/s43019-020-00080-1 (PMC7667791; doi:10.1186/s43019-020-00080-1)
Supplement: Supplementary file 1 — Additional file 1. Modified Coleman Criteria used of quality assessment of studies. [file 43019_2020_80_MOESM1_ESM.docx]

| Additional file 1. Modified Coleman Criteria used of quality assessment of studies | | |
| --- | --- | --- |
|  | Description | Score |
| Part A |  |  |
| 1. Study size (numbers of TKAs) | >100 | 10 |
|  | 51-100 | 7 |
|  | 11-50 | 4 |
|  | <10 | 0 |
| 2. Mean follow-up (y) | >20 | 10 |
|  | 11-20 | 7 |
|  | 6-10 | 4 |
|  | 2-5, not stated or unclear | 0 |
| 3. Type of study (methodology) | Randomized controlled trial | 10 |
|  | Prospective cohort study | 7 |
|  | Mixed cohort study | 4 |
|  | Retrospective cohort study | 0 |
| 4. Diagnostic certainty (underlying etiology: eg. degenerative/ inflammatory/post-trauma) | All osteoarthritis | 10 |
|  | >80% osteoarthritis | 5 |
|  | <80% osteoarthritis or unknown | 0 |
| Part B |  |  |
| 1. Outcome criteria | Clearly defined outcome (s) | 3 |
|  | Timing of outcome assessment clearly stated | 3 |
|  | PROMs used | 3 |
|  | Overall satisfaction | 3 |
|  | Radiological assessment | 3 |
| 2. Procedure for assessing outcomes | Clearly defined method | 2 |
|  | Objective assessment | 2 |
|  | Multiple/independent observers | 2 |
| 3. Description of subject selection from population | Inclusion criteria reported and unbiased | 4 |
|  | Recruitment rate reported >80% | 3 |
|  | Recruitment rate reported <80% | 2 |
|  | Recruitment not reported | 0 |
| 4. Post-operative rehabilitation | Well described | 6 |
|  | Not adequately described | 3 |
|  | Protocol not reported | 0 |
| 5. Surgical technique | Single prosthesis used | 3 |
|  | Surgical approach stated and same throughout | 3 |
|  | Fixation method stated | 3 |
| 6. Complications recorded | All with explanations | 8 |
|  | Selected complications reported | 4 |
|  | Incomplete record | 2 |
|  | None | 0 |
| 7. Revisions recorded | Method of failure (s) | 3 |
|  | Time to failure (s) | 3 |
|  | No failures in study | 2 |
